# Measuring patient experiences in Fabry disease: validation of the Fabry-specific Pediatric Health and Pain Questionnaire (FPHPQ)

**DOI:** 10.1186/1477-7525-10-116

**Published:** 2012-09-20

**Authors:** Uma Ramaswami, Donald E Stull, Rossella Parini, Guillem Pintos-Morell, Catharina Whybra, Gisela Kalkum, Marianne Rohrbach, Mireia Raluy-Callado, Michael Beck, Wen-Hung Chen, Ingela Wiklund

**Affiliations:** 1 The Willink Biochemical Genetics Unit, Genetic Medicine, St Mary’s Hospital, Manchester, UK; 2 RTI Health Solutions, Manchester, UK; 3 San Gerardo Hospital, Università Milano Bicocca, Monza, Italy; 4 Department of Pediatrics, “Germans Trias i Pujol” Hospital, Universitat Autònoma de Barcelona, Badalona, Spain; 5 Department of Pediatrics, University Medical Center, University of Mainz, Mainz, Germany; 6 University Children's Hospital, Zurich, Switzerland; 7 United BioSource Corporation, London, UK; 8 United BioSource Corporation, Bethesda, MD, USA

**Keywords:** Fabry disease, Enzyme replacement therapy, Children, Paediatric Health and Pain Questionnaire, Psychometrics validation

## Abstract

**Introduction:**

Common symptoms for children with Anderson-Fabry Disease (FD) such as acroparaesthesia and gastrointestinal manifestations can only be objectively assessed in patients using a valid instrument. To date, no such instrument exists.

**Methods:**

A preliminary 40-item measure of symptoms and experience with FD, the Fabry-specific Paediatric Health and Pain Questionnaire (FPHPQ) was developed, but lacked a formal assessment of its measurement properties. The FPHPQ was used in the Fabry Outcome Survey (FOS), a registry for all patients with a confirmed diagnosis of FD who are receiving agalsidase alfa, or are treatment naïve and who are managed by physicians participating in FOS. After an item analysis to explore how items performed and combined into domains, a battery of psychometric analyses was performed to assess the measurement properties of this new instrument.

**Results:**

Eighty-seven children (ages 4-18 years) completed the questionnaire. Twenty-three items in three subscales of the questionnaire emerged: pain associated with heat or exertion, pain associated with cold, and abdominal pain and fatigue symptoms. Internal consistency reliability for all three subscales was good (Cronbach alpha ≥ 0.84). Reliability was equally high for all age groups (4-7, 8-12, and 13-18). Test-retest reliability was high for all three subscales (intraclass correlation coefficient ≥ 0.74). Construct validity was demonstrated by moderate correlation with brief pain inventory (BPI), KINDL, and EQ-5D. Known group validity showed all subscales were able to discriminate between Fabry disease severity groups as classified by above or below median of the FOS MSSI (Mainz Severity Score Index) grade. The heat or exertion subscale was responsive to change in symptoms between responders and non-responders as defined by change in EQ-5D index scores between the first and second visit.

**Conclusions:**

Preliminary results indicate that the measurement properties of FPHPQ are valid and reliable for assessing patient-reported symptoms of FD. The questionnaire could be a useful tool for clinicians to understand the progression of disease and monitor treatment effects. FPHPQ will be further validated and refined as the FOS registry is continuously adding more patients.

## Introduction

Anderson-Fabry disease (FD) is a rare condition, but the second most common among the lysosomal storage diseases (LSD) and the only X-linked sphingolipidosis [1,2]. It is an inherited disorder caused by a deficiency of alpha-galactosidase A (GLA) that results in a slowly progressive disease with premature death in adult males and some females due to cardiac, renal or central-nerve-system (CNS) events [2]. FD is rare with an estimated incidence of 1 in 40,000 to 60,000 males, with clinical heterogeneity in female patients [3-6]. The disease typically begins in childhood and can be diagnosed by measuring the level of alpha-galactosidase activity; however, this may be misleading in female heterozygotes probably due to the random nature of X-inactivation [7]. Molecular analysis of the GLA gene is the most accurate method of diagnosis, and many mutations which cause the disease have been noted.

Characteristic features of FD include episodes of neuropathic pain [8]. Other symptoms may include fatigue, nausea, dizziness, gastrointestinal symptoms such as diarrhoea, a decreased ability to sweat, angiokeratoma, cornea verticillata and hearing impairment may also occur in childhood.

In rare conditions such as FD, close and prospective monitoring of as many patients as possible is important to gain a better understanding of the natural history of the disease and the rate of disease progression. Additionally, to assess the impact of treatments such as enzyme replacement therapy (ERT), a valid disease-specific measure is required. Because no instrument to assess the typical manifestations of the disease existed, the Fabry-specific Paediatric Health and Pain Questionnaire (FPHPQ) was developed and has been used to address the most commonly reported symptoms experienced by children with FD followed in a Fabry registry – the Fabry Outcome Survey (FOS; sponsored by Shire Human Genetics Therapies).

The preliminary FPHPQ was a 40-item questionnaire on children’s symptoms and experience with FD developed by the FOS Paediatric Working Group - an international group of dedicated and experienced clinicians. The FOS Paediatric Working Group set out to develop a tool that would both identify disease burden in children that affected their quality of life but also in the future once validated, could be used to monitor treatment effects. The questions were specifically chosen based on what the clinical experts knew at the time of the subjective early clinical manifestations of FD.

FOS is the world's most comprehensive database on medical outcomes of patients with FD. FOS is designed as an international multi-centre, open-label registry [9]. It is open to all patients on, or candidates for ERT with agalsidase alfa. Patients with FD entered into the database but not receiving ERT are also followed in order to gain insight into the natural history of the condition. The principal aim of FOS is to collect and disseminate information about the long-term course of the disease, especially in patients treated with agalsidase alfa. In order to guide treatment, relevant data recorded on the FOS database are being made available to the treating physician on an individual patient basis. Questions to the database aimed at helping FOS physicians in their routine clinical practice and patient management to be handled swiftly [9].

The purpose of this study is to present the psychometric validation of the FPHPQ, as well as to explore the underlying concepts measured and their dimensionality.

## Methods

Data were obtained from the FOS. The inclusion criteria were patients of both sexes with a confirmed diagnosis of FD, who were on, or were candidates for, ERT with agalsidase alfa. The exclusion criteria were patients who were treated with an ERT other than agalsidase alfa or were actively enrolled in blinded trials so not eligible for enrolling in the FOS. FOS was performed in accordance with the recommendations of Declaration of Helsinki. Where necessary, participating centres followed their respective national and/or local regulations regarding Ethics Committee/Institution Review Board requirements.

An internet-based application was used for entering data into the FOS database. Data were entered into the database by the responsible FOS physician or designee. The patients self-reported the survey instruments including the FPHPQ, and responses were entered into the database through the internet application. The cut-off date for this analysis was April, 2010.

Patients were followed as long as the physician found it appropriate. Only data collected during normal routine examinations were requested. At screening/baseline visit, age and gender, comorbidities, concomitant medication, and FOS - Mainz Severity Score Index (FOS MSSI) [10,11] were collected. FPHPQ and other patient reported outcome (PRO) instruments were administered at subsequent visits. Given that FOS is an international, multi-centre, open survey, the PRO questionnaires were available to participating patients in their respective languages for most of the countries. Because FD is a rare disease, it was necessary to pool data of multiple languages to be able to include patients with a wide range of disease severity and to ensure a sufficient sample size for data analysis. Taking into consideration the language difference, efforts have been made to harmonize these questionnaires so that answers can be interpreted in international analyses. In addition, the translations were reviewed by bi-lingual clinical experts with particular scrutiny of wording appropriate for children. These patient-completed questionnaires are briefly described below.

### Fabry-specific Paediatric Health and Pain Questionnaire (FPHPQ)

The FPHPQ assesses disease specific symptoms such as sweating, pain, dizziness and tiredness, heat and cold intolerance, swollen eyelids, gastrointestinal symptoms, feeling thirsty, difficulty hearing, ringing or buzzing noise in the ears, and ability and enjoyment to participate in sports. The FPHPQ measures the frequency of each specific symptom using a 5-point Likert scale (always, often, sometimes, seldom, and never), plus one item that measures the pain intensity using a 0-10 numeric rating scale. In addition, two items require numeric responses about the number of times experiencing onset of pain and the number of school days missed. Finally, there are two items with ‘yes’ or ‘no’ response asking about difficulty in hearing and other problems not mentioned. There are three separate age-specific versions for children aged 4-7, 8-12 and 13-18 years with questions that are phrased age appropriately. The parents of children aged 4-7 complete the FPHPQ; children aged 8 and above complete the questionnaire themselves. Translations of the FPHPQ are available in Spanish, Swedish, English, Norwegian, French, Dutch, Italian, and German.

### FOS - Mainz Severity Score Index (FOS MSSI)

The MSSI is a clinical scoring system developed to assess the severity of signs and symptoms of FD and to monitor the progress of individual patients during ERT. The FOS MSSI is an adaptation of the MSSI scoring system for use in FOS and has been shown to be a useful and valid tool to evaluate disease severity and progression in adult patients with FD [10,11].

### The Brief Pain Inventory (BPI)

The BPI questionnaire is a validated tool developed to capture the “sensory” dimension of pain on a 0–10 numerical rating scale. It includes four pain items to capture the variability of pain over time and seven items assessing the “reactive” dimension of pain (interference with daily function) [12,13]. Initially developed to assess pain related to cancer, the BPI has shown to be an appropriate measure for pain caused by a wide range of clinical conditions including FD [14,15].

### The KINDL

The KINDL questionnaire is a generic validated questionnaire developed for use in children and adolescents to evaluate the impact of health conditions on everyday living and quality of life (QOL) [16,17]. The KINDL questionnaire comprises 24 items using a 5-point Likert scale (never, seldom, sometimes, often, all the time) and includes six subscales depicting physical well-being, emotional well-being, self-esteem, family, friends, and everyday functioning (school or nursery school/kindergarten).

### The EQ-5D

The EuroQol valuation instrument, or EQ-5D, is an international standardized instrument designed to measure health and to allow the elicitation of patient and/or general population preference values for a wide range of standardized health states [18].

The EQ-5D self-report questionnaire defines health in terms of five dimensions: mobility, self-care, usual activities, pain or discomfort, and anxiety or depression. It also includes the respondents’ perception of their overall health on the visual analogue scale (EQ VAS), where 0 and 100 denote the worst and the best health states, respectively [19]. The EQ-5D has been used in previous FD studies [20,21].

#### Statistical analysis: item analysis, reliability, validity, and responsiveness

A battery of psychometric analyses, described below, was performed to a) identify poorly performing items and b) to assess the measurement properties of the instrument once these items were excluded. All analyses were conducted in several iterative steps with the use of Stata/MP Ver. 11.0 [22], with the exception of the factor analysis which was performed using Mplus Ver. 6.0 [23].

First, the distributional characteristics of each of the items (e.g., % missing, % at floor and ceiling, skewness and kurtosis) were reviewed to identify poorly performing items. Items were flagged for potential exclusion if the distribution of responses was highly skewed or kurtotic or they showed a floor or ceiling effect (minimum/maximum response > 67% of patients). Exploratory factor analysis (EFA) was also used as part of the initial item analysis to help identifying overlapping or redundant items. Items with factor loadings <0.34 (i.e., less than 10% of variance explained) on their primary subscale were flagged for possible deletion. Yes/no items were excluded as these violate the distributional assumptions of EFA. The final decision of item inclusion or exclusion was based on all of the psychometric analyses, and input from the clinical and instrument development experts.

After the exclusion of items from above, a follow-up EFA was conducted to investigate the dimensionality of the items retained. The purpose of the follow-up EFA was to identify items that cluster into conceptually related subscales as well as items that lacked or had weak relationships with the other items and did not belong to a specific subscale. Three types of rotation were consistently used to help interpret simple structure (quartimax and varimax, which are orthogonal rotations; and oblique, which allows correlations between subscales). A scoring algorithm was developed following the final selection of the items to be included in the instrument.

Next, the psychometric properties of the FPHPQ subscales that emerged from the follow-up EFA were examined. The psychometric analyses included descriptive statistics of the subscales and their internal consistency reliability, test-retest reliability, validity and responsiveness to change. The internal consistency of the FPHPQ subscales was assessed using Cronbach’s formula for coefficient alpha, and whenever Cronbach’s alpha exceeded 0.70 it was considered having good internal consistency reliability. The analysis for test-retest reliability of the FPHPQ involved calculating the intraclass correlation coefficient (ICC) based on data of those who reported no change on the patient global impression of change (PGIC) from first to second visit. An ICC of >0.70 among stable subjects is considered acceptable to demonstrate test-retest reliability [24].

Validity refers to the extent to which the instrument measures what it is intended to measure [25]. To demonstrate construct validity, the score of an instrument should be correlated with scores of other validated instruments that measure similar concepts. The construct validity of the FPHPQ subscales was evaluated through correlation with the scores of the criterion measures BPI, KINDL, and EQ-5D. Rank order correlations were calculated and were expected to be ≥ 0.30 and statistically significant. Known-group validity is the extent that the average scores of the FPHPQ are significantly different among groups of FD patients with different level of disease severity. Analysis of variance (ANOVA) was used to examine the FPHPQ scores by the FOS MSSI grades.

Responsiveness (or ability to detect change) refers to the extent that the score of an instrument reflects the changes in the patient’s condition. The change of the score should be in sync with the direction of the condition: improving, worsening or unchanged. Patients with improving condition should have significantly better scores than the patients with worsening or unchanged condition. The magnitude of the score change can also be used to guide score interpretation. Change measured as standardized effect sizes with 0.20 denotes a small and not clinically relevant change; 0.50 denotes a moderate and clinically relevant change; and 0.80 or above denotes a large and clearly relevant change [26]. A preliminary assessment of responsiveness of the FPHPQ was made by examining the amount of change in FPHPQ subscale scores between responders versus non-responders with responder defined as EQ-5D score increased 0.1 point from first to second visits, and separately with responder defined as BPI average pain and worst score rating decreased 20%, also from first to second visits.

## Results

The FOS was conducted in 19 countries countries that included Argentina, Australia, Austria, Belgium, Canada, Czech Republic, France, Germany, Hungary, Italy, Japan, the Netherlands, Norway, Slovenia, Spain, Sweden, Switzerland, United Kingdom, and United States. Out of the 299 children registered in FOS at the cut-off time of this study, responses from 87 children (aged 4-18 years) that completed the questionnaire in eight of the countries were used for these analyses. Number of patients, response rate for the FPHPQ, and average study period in FOS are shown in Table 1.

**Table 1:** Number of patients at data cut-off date, response rate, and average study period by country

	**Number of Patients at Data Cut-off Date (Total N = 299)**		**Patients Completed FPHPQ (Total N = 87)**		**Response Rate (%)**	**Average Study Period (Years)**
Country	**N**	**%**	**N**	**%**		
Argentina	17	6%	12	14%	71%	2.44
Australia	8	3%	6	7%	75%	4.19
Belgium	6	2%	2	2%	33%	5.11
Canada	17	6%	7	8%	41%	4.06
Germany	53	17%	24	28%	45%	3.79
Spain	17	6%	9	10%	53%	2.34
Italy	25	8%	9	10%	36%	5.46
United Kingdom	40	13%	18	21%	45%	3.40

Demographic and clinical characteristics of the 87 children included in this analysis, displayed by the three age cohorts, are shown in Table 2. More than 50% of the sample was in the oldest age group (13-18 years), and the gender distribution was even. Table 3 displays the descriptive statistics for the other PRO instruments: BPI, KINDL and EQ-5D at first visit.

**Table 2:** Patient characteristics

	**All ages (N = 87)**		**Age group 4-7 (N = 10)**		**Age group 8-12 (N = 30)**		**Age group 13-18+ (N = 47)**	
	**N**	**%**	**N**	**%**	**N**	**%**	**N**	**%**
**Gender**								
Female	43	49%	4	40%	16	53%	23	49%
Male	44	51%	6	60%	14	47%	24	51%
								**Treated with Agalsidase Alfa**								
No	37	43%	7	70%	17	57%	13	28%	
Yes	50	57%	3	30%	13	43%	34	72%	
**FOS MSSI**									
Mild (<20)	81	93%	10	100%	27	90%	44	94%	
Moderate (20-40)	3	3%	0	0%	1	3%	2	4%	
Missing	3	3%	0	0%	2	7%	1	2%	

**Table 3:** BPI, KINDL, and EQ-5D scores

**(N = 87)**	**Mean**	**St. Dev**	**% Missing**
**BPI Scores**			
Worst pain last 24 hours	2.53	2.86	63%
Least pain last 24 hours	0.75	1.44	63%
Average Pain	2.03	2.09	63%
Pain right now	0.72	1.71	63%
Average interference score	0.76	1.47	57%
**KINDL Scores**			
Physical health	1.46	0.68	46%
General feeling	1.22	0.48	46%
Personal feeling	2.58	0.99	47%
Family	2.38	0.70	47%
Friends	2.88	0.89	48%
School	2.01	0.91	51%
Stayed in hospital	0.37	0.49	53%
Illness	1.11	0.69	57%
**EQ-5D Scores and Utilities**			
Mobility	1.11	0.31	46%
Self care	1.00	0.00	46%
Usual activities	1.11	0.31	46%
Pain/discomfort	1.53	0.55	46%
Anxiety/discomfort	1.09	0.28	46%
EQ-5D utility	1.00	0.00	46%
EQ-5D VAS	1.11	0.31	46%

### Item analysis

Among the original 40 items in the FPHPQ, 27 items were retained after investigation of descriptive statistics analysis and the initial EFA. The main reasons for deletion were high proportion (>80%) of missing responses or a high proportion (>67%) of floor effects (4 items) indicating low relevance of the item, and high skewness or kurtosis (2 items). Based on the initial EFA, items with high uniqueness (high error) and/or communality estimates (multiple R^2^) below 0.3 were also deleted (3 items). Items including binary responses (Yes/No) were also excluded from the initial EFA (4 items).

These 27 items retained were classified into symptom-related (23 items) and outcome-related (4 items). Symptom-related items included items asking about pain, burning sensation, tiredness, diarrhoea, and bloating in various conditions. The outcome-related items included items that assess if children like playing sports, participate in sports, get tired when playing sports, and get more tired compared to friends; they were analyzed separately and were used for descriptive purposes. Only the symptom-related items were included in the follow-up EFA to identify specific subscales within the FPHPQ.

EFA on the 23 symptom-related items identified was conducted using three different rotations: orthogonal (varimax and quartimax) and oblique (oblimin) rotations. Factor loadings from oblique rotation are shown in Table 4. Results suggested that the 23 symptom-related items clustered into three subscales: pain associated with heat or exertion; pain associated with cold; and abdominal pain and fatigue symptoms. The eigenvalues for these subscales were 8.90, 3.10 and 1.53, respectively, which accounted for 59% of the total variance.

**Table 4:** EFA – Symptoms items, oblique rotation*

**Item**		**Pain Associated with Heat or Exertion**	**Pain Associated with Cold**	**Abdominal Pain & Fatigue**	**Uniqueness**
23d	Get burning sensations in fingers and toes when playing?	**0.94**	−0.09	0.04	0.16
22d	Get shooting pains in fingers and toes when playing?	**0.90**	−0.02	0.01	0.21
22b	Get shooting pains in fingers and toes when it is hot?	**0.81**	0.22	−0.21	0.20
23a	Get burning sensations in fingers and toes when fever?	**0.80**	−0.09	0.15	0.35
22a	Get shooting pains in fingers and toes when fever?	**0.79**	−0.09	0.22	0.32
2a	In hot surroundings, pain?	**0.77**	0.26	−0.22	0.22
20	Upsetting pain?	**0.72**	−0.06	0.30	0.35
23b	Get burning sensations in fingers and toes when it is hot?	**0.70**	0.34	−0.11	0.24
26	Number of times of sudden onset of pain?	**0.49**	0.19	0.21	0.54
22c	Get shooting pains in fingers and toes when it is cold?	0.06	**0.82**	0.09	0.23
3a	In cold surroundings, pain?	0.12	**0.72**	−0.12	0.43
23c	Get burning sensations in fingers and toes when it is cold?	0.26	**0.66**	0.02	0.33
25	Muscle pain?	−0.09	**0.60**	0.37	0.44
24	Joint pain?	0.00	**0.54**	0.33	0.50
7	After eating, bloated or full?	0.03	0.08	**0.73**	0.42
6	Pain gets worse with eating?	0.20	−0.12	**0.71**	0.45
9	Diarrhea?	0.05	0.05	**0.67**	0.51
5	Pain in the tummy?	0.09	0.01	**0.63**	0.57
10	Tummy pain with diarrhea?	−0.11	0.35	**0.53**	0.54
13	Frequently feel thirsty?	−0.18	0.33	**0.51**	0.60
12	Often wake up during the night to go to toilet?	−0.02	0.13	**0.48**	0.73
2c	In hot surroundings, tiredness?	0.24	0.23	**0.46**	0.53
3c	In cold surroundings, tiredness?	0.15	0.32	**0.40**	0.59

* Other rotations yielded nearly identical results.

#### Scoring algorithm

For the validation purposes of the FPHPQ, the five graded Likert-type response options were transformed into numeric variables in the following way: 0 = Never, 1 = Seldom, 2 = Sometimes, 3 = Often, 4 = Always. Then, each of the subscales was scored by computing the raw sum of responses to each of its items, with equal weight to all the items. A higher subscale score reflects more severe symptoms, or worse health.

### Reliability, validity, and responsiveness

The Cronbach alphas were 0.94, 0.85, and 0.85 for pain associated with heat or exertion, pain associated with cold, and abdominal pain and fatigue subscales, respectively, showing good internal consistency reliability across all age groups. Scores between first and second visits were used to assess the test-retest reliability. Time between first and second visit varied: less than a month for more than half of the children (52.63%), between one and two months for more than a third (36.84%) and between two and three months (10.53%) for the rest. The ICCs were 0.90, 0.74, and 0.77 for pain associated with heat or exertion, pain associated with cold, and abdominal pain and fatigue subscales, respectively, showing good test-retest reliability across all age groups.

Tests for construct validity indicated that each subscale measured unique patient symptom experiences and was correlated with other instruments in expected ways, as shown in Table 5. Correlations between the three subscales and the EQ-5D index score were in the moderate range (0.31 to -0.43) and statistically significant at p < 0.05. The correlation between the pain associated with cold, and abdominal pain and fatigue subscales with the BPI pain interference score was in the moderate range (0.33 and 0.35, respectively) and statistically significant at p < 0.05. However, the correlation between the pain associated with heat or exertion subscale and BPI pain interference score was a little lower at 0.25. Likewise, the correlation between the pain associated with cold, and abdominal pain and fatigue subscales with the BPI worst pain item were in the moderate range (0.39 and 0.32, respectively) and statistically significant at p < 0.05. Still, the correlation between the pain associated with heat or exertion subscale and BPI worst pain item was 0.22. Finally, the three FPHPQ subscales were modestly correlated with the KINDL physical health (0.22 to 0.43), personal feelings (-0.35 to 0.47), friends (-0.25 to -0.43), and illness (0.27 to 0.44) suggesting that the symptom components of the FPHPQ are related to the physical health and social domains measured by the KINDL.

**Table 5:** Construct validity – FPHPQ correlations with EQ-5D, BPI, and KINDL

	**FPHPQ**		
	**Pain Associated with Heat or Exertion**	**Pain Associated with Cold**	**Abdominal Pain & Fatigue**
**EQ-5D Utility**	−0.43*	−0.31*	−0.35*
**BPI Pain Interference Score**	0.25	0.33*	0.35*
**BPI Pain Intensity**			
Worst Pain	0.22	0.39*	**0.32**
Least Pain	0.21	0.12	0.19
Average Pain	0.01	0.04	0.06
Pain Right Now	−0.02	0.07	−0.04
**KINDL**			
Physical Health	0.22	0.39*	0.43*
General Health	−0.11	0.07	0.16
Personal Health	−0.35*	−0.47*	−0.29
Family	0.08	−0.20	−0.17
Friends	−0.25	−0.43*	−0.27
School	−0.23	−0.03	−0.05
Illness	0.44*	0.32*	0.27

Correlations > 0.30 are in bold.

* indicates correlation coefficient is significant at 5% level of confidence.

To assess known-group validity, FOS MSSI grade was used to classify patients into two groups for comparison according to disease severity. Using the FOS MSSI between 20-40 as the criterion, only three patients were classified as moderate severity versus 81 patients as mild severity (FOS MSSI < 20), so the sample size of the moderate severity group was too small for the statistical comparison. Therefore, for this study the median FOS MSSI of the sample (median = 10.5) was used to classify the patients into above and below median groups. The ANOVA results in Table 6 show significant difference for all three FPHPQ subscale average scores across the above and below median groups. The known-group validity of the FPHPQ was therefore demonstrated with the significant difference between the subscale average scores of the two groups.

**Table 6:** Known-groups validity of the FPHPQ subscales: ANOVA by FOS MSSI severity groups at baseline

**FPHPQ Subscales**	**N**	**FPHPQ score Mean (SD)**	**F-test*P*Value**
**Pain Associated with Heat or Exertion**			0.0261
Severity score below median	35	9.7 (10.49)	
Severity score above median	39	15.2 (10.28)	
**Pain Associated with Cold**			0.0175
Severity score below median	32	6.9 (7.05)	
Severity score above median	36	10.8 (5.94)	
**Abdominal Pain & Fatigue**			0.0341
Severity score below median	36	1.9 (2.67)	
Severity score above median	40	3.5 (3.78)	

Responsiveness of FPHPQ subscale scores was demonstrated by examining whether the average subscale score change from first to second visit was significantly different between the responder and the non-responder groups. Three definitions of responder were used: 1) responder as EQ-5D index score increased 0.1 point from first to second visits; 2) responder as BPI average pain rating decreased 20% from first to second visits; 3) responder as BPI worst pain rating decreased 20% from first to second visits. Based on the EQ-5D definition, among the 87 patients, 10 were identified as responders and 31 as non-responders, the rest with missing data. The intervals between the two visits ranged from 3 to 56 months. Using ANOVA, as shown in Table 7, the average score changes between the responder and non-responder groups were significantly different for pain with heat and exertion subscale, but were not significantly different for pain with cold or for abdominal pain and fatigue subscale. Based on the BPI average pain definition, there were only 5 responders versus 16 non-responders, and the rest with missing data. The intervals between the two visits ranged from 3 to 46 months. Based on the BPI worst pain definition, there were only two responders versus 19 non-responders, and the rest with missing data. Responsiveness using the BPI average pain or worst score to define responders was not assessed owing to the small sample size.

**Table 7:** Responsiveness for the FPHPQ subscales using EQ-5D definition

**FPHPQ Subscales**	**Responder – EQ-5D Score Change > 0.1 from Visit 1 to 2**		**Non-responder – EQ-5D Score Change < 0.1 from Visit 1 to 2**		**F-Test*P*Value**
	**N**	**Mean (SD)**	**N**	**Mean (SD)**	
Pain Associated with Heat or Exertion	10	−3.5 (6.40)	31	2.4 (6.80)	0.0197
Pain Associated with Cold	10	−0.1 (1.94)	31	0.3 (2.56)	0.6725
Abdominal Pain & Fatigue	10	0.7 (3.25)	31	−0.5 (3.61)	0.3600

## Discussion

Establishing the psychometric properties of a measure, especially a new measure, is critical especially when this is used to monitor patients over time [24,25,27]. In FD, a rare condition with a low prevalence, no valid and reliable measure for monitoring patient progress under treatment existed [7-9,28]. Well established psychometric properties are a prerequisite for careful monitoring of patient health, for scientific publication and successful regulatory submissions. By careful item reduction, a more efficient measure can be created, which has benefits for use in clinical practice or longitudinal studies to evaluate treatment benefits.

The item analysis of FPHPQ showed that 27 of the 40 items met the psychometric property requirement to be retained in the instrument. EFA results showed a potential 3-factor structure for the 23 symptom items: pain associated with heat or exertion, pain associated with cold, and abdominal pain and fatigue symptoms. All three subscales showed excellent internal consistency reliability and the test-retest reliability. Reasonable patterns of relationships for construct validity of all three subscales were shown, especially relationships between EQ-5D and BPI pain interference score. In addition, the three FPHPQ subscales were correlated with the physical health and social domains as measured by the KINDL.

Known-group validity was demonstrated by significant difference in average scores for all three subscales between groups divided by FOS MSSI median score. A small number of responders (n = 10) was identified based on the definition of EQ-5D score increase of 0.1. The major limitation of the study is the small number of patients involved that limited the feasibility of some psychometric assessments, specifically confirmatory factor analysis (CFA) to confirm the factor structure of 3 factors and responsiveness to determine the FPHPQ’s ability to detect change in patients clinical condition. Because FD is a rare disease, it was necessary to pool the data from various countries to have sufficient sample size. Even so, the sample size of the pooled data was still not large enough to take into consideration of the possible language differences between the countries; which is another limitation of this study. In the future, when the sample size allows, FPHPQ should be evaluated for existence of language differences. The study was further limited by the widely variable interval between physician visits and the high percentage of missing data. These hampered the statistical comparisons and the generalization of the study results. FD research would benefit a lot from good data collection and physicians should be encouraged to maintain regular patient follow-up visits and adhere to the FOS registry guidance. The FOS registry is continuously adding patients, so current results should be considered as preliminary and need to be validated again once a larger sample of FD patients has been accumulated in the registry [9,29].

Exploration of further applications of the use of the FPHPQ should be considered. Because of the rarity of the condition there is a need to improve the understanding of FD and facilitate early detection to avoid delays in its diagnosis and treatment [9,28]. Performing further item reduction to achieve a streamlined screening instrument for use in clinical practice to detect discrete but typical symptoms indicative of FD would be an important future objective. Additionally, the analysis of potential differences by subgroups, such as by age cohort or between countries, should be considered in the future [29].

In conclusion, preliminary results of psychometric analyses of the FPHPQ are very satisfactory as the three subscales (composed of 23 items) exhibit very satisfactory measurement properties in terms of reliability and validity. The large score difference for responsiveness in the pain associated with heat or exertion subscale indicates that this subscale reflects an important aspect of FD from the patient perspective that captures salient patient experiences. The FPHPQ questionnaire is a useful tool for clinicians to understand the severity and progression of FD in childhood and to document treatment benefits over time.

## Abbreviations

(GLA): alpha-galactosidase; (ANOVA): Analysis of variance; (BPI): Brief Pain Inventory; (CFA): Confirmatory factor analysis; (CNS): Central-nerve-system; (EFA): Exploratory factor analysis; (EQ VAS): Visual analogue scale; (ERT): Enzyme replacement therapy; (FD): Anderson-Fabry disease; (FOS): Fabry Outcome Survey; (FOS MSSI): FOS-Mainz Severity Score Index; (FPHPQ): Fabry Paediatric Health and Pain Questionnaire; (ICC): Intraclass correlation coefficient; (LSD): Lysosomal storage diseases; (PGIC): Patient global impression of change; (PRO): Patient reported outcome; (QOL): Quality of life.

## 

* Note: This work was conducted while Dr. Stull was at United BioSource Corporation, Bethesda, MD. RTI-HS was not affiliated in the development of this work.

## Competing interests

Uma Ramaswami has received travel and research grants and honoraria for invited lectures from Shire HGT, Genzyme and Actelion. Gisela Kalkum has received travel and congress grants from Shire HGT. Donald Stull is employed by RTI Health Solutions (RTI-HS) but during the development of this manuscript he was employed by United BioSource Corporation (UBC). Both companies provide consulting and other research services to pharmaceutical, device, government, and non-government organizations. In his salaried positions, he works with a variety of companies and organizations. He receives no payment or honoraria directly from these organizations for services rendered. Guillem Pintos-Morell has received honoraria, travel, and research grants from Shire HGT. Michael Beck has received honoraria, travel support, and unrestricted grants from Shire, Genzyme, Biomarin, and Actelion. Catharina Whybra has received honoraria, travel, and research grants from Shire HGT. Rossella Parini has received travel and congress grants and honoraria for oral presentations from Shire HGT and Genzyme. Marianne Rohrbach has received honoraria, travel, and research grants from Shire HGT. Ingela Wiklund is employed by United BioSource Corporation (UBC), which provides consulting and other research services to pharmaceutical, device, government, and non-government organizations. In this salaried position, Ingela Wiklund works with a variety of companies and organizations. She receives no payment or honoraria directly from these organizations for services rendered. Mireia Raluy-Callado is employed by United BioSource Corporation (UBC), which provides consulting and other research services to pharmaceutical, device, government, and non-government organizations. In this salaried position, Mireia Raluy works with a variety of companies and organizations. She receives no payment or honoraria directly from these organizations for services rendered. Wen-Hung Chen is employed by United BioSource Corporation (UBC), which provides consulting and other research services to pharmaceutical, device, government, and non-government organizations. In this salaried position, Wen-Hung Chen works with a variety of companies and organizations. He receives no payment or honoraria directly from these organizations for services rendered.

## Authors’ contributions

UR, RP, GPM, CW, GK, MR, and MB contributed to conception and design of the study, acquisition of data and interpretation of data; DES, MRC, WHC, and IW contributed to analysis and interpretation of data. All authors contributed to drafting the article or reviewing and revising it critically for important intellectual content; all authors approved the final version to be published. IW attests that the authors had access to all the study data, takes responsibility for the accuracy of the analysis, and had authority over manuscript preparation and the decision to submit the manuscript for publication.

## Funding source

This manuscript was funded by Shire AG.
